# Evaluation of Dermal Toxicity and Wound Healing Activity of *Cnestis ferruginea* Vahl ex DC

**DOI:** 10.1155/2022/5268613

**Published:** 2022-05-23

**Authors:** Akosua Dufie Ankomah, Yaw Duah Boakye, Theresah Appiah Agana, Vivian Etsiapa Boamah, Paul Poku Sampene Ossei, Francis Adu, Christian Agyare

**Affiliations:** ^1^Department of Pharmaceutics, Faculty of Pharmacy and Pharmaceutical Sciences, Kwame Nkrumah University of Science and Technology, Kumasi, Ghana; ^2^Department of Pathology, School of Medical Sciences, Kwame Nkrumah University of Science and Technology, Kumasi, Ghana

## Abstract

*Cnestisferruginea* is a tropical plant, which is traditionally used in the treatment and management of various conditions including skin infections and wounds. The aim of this study was to investigate the dermal toxicity and wound healing potential of *C. ferruginea*. Ten millimeter full-thickness mucosal wounds were created on the dorsal midportion of the Sprague Dawley rats. Wounds were treated with 10, 5, and 2.5% w/w aqueous creams, prepared from the methanol extract of the root bark of *C. ferruginea* (CFM). The wound tissues were harvested on day 21 for histology studies. Compared with the untreated group, 10, 5, and 2.5% w/w CFM-treated wounds significantly reduced the wound size over the study period (*P* < 0.0001). Tissue histology revealed a healed wound with well-regenerated collagen and skin appendages with no pus cells. A skin irritation test was conducted on CFM, as well as the dermal toxicity of CFM was determined in the repeated dose and acute dermal toxicity bioassays. These tests revealed that CFM showed no toxic effect on the skin and showed that CFM was not a skin irritant. *C. ferruginea* exhibited wound healing activity, which gives credence to its folkloric use.

## 1. Introduction

Every year around the world, many people get wounded as they are often inevitable occurrences[1]. Apart from the morbidity, disability, and reduced quality of life it imposes on patients, it also creates an economic challenge to the individual and the country at large [2]. Wounds are created when there are interruptions in the integrity of the skin and muscles, as well as bones [3]. It may be attributed to causes such as burns, gunshots, falls, surgical procedures, deleterious skin infection, or by other basal conditions [4]. Wounds appear to be the third most frequent nosocomial infection and unfortunately for developing and resource-constrained countries such as Ghana and others, traumatic and surgical site infections cause high morbidity and death rates [5].

When wounds occur, the body naturally regenerates the skin tissues through the wound healing process [6]. The process of wound healing includes different overlapping events and is categorized into four phases namely homeostasis, inflammation, proliferation, and remodeling phases [7]. Medicinal plants are used in managing acute and chronic wounds in many traditional medicine practice areas in the world, and this practice is a common one [8]. Undoubtedly, these plants have become a great source of wound healing agents to tap into [9].

*Cnestis ferruginea* of the family Connaraceae is a common plant found in the tropics [10]. Traditionally, it is used to treat migraine (root), headache (root), toothache (root), sinusitis (root), constipation (leaf or root), and conjunctivitis (fruit) [10]. It is used in the management of skin diseases and also in the management of wounds though this has not been scientifically studied [11]. Scientifically, *C. ferruginea* has been proven to exhibit several biological activities. The hydroethanolic leaf extract of *C. ferruginea* has been studied to have wound healing activity [12]. The methanol root extract has been reported to exhibit analgesic and anti-inflammatory properties [13, 14]. The methanol and ethyl acetate leaf extracts of *C. ferruginea* have been reported to possess hypoglycaemic properties [15]. The aqueous, ethanol, and petroleum ether fractions of the leaf, stem, bark, and root extracts of *C. ferruginea* exhibit antioxidant activity [16]. The aqueous root extract of *C. ferruginea* has been reported to possess antistress potentials [17]. Also, it is worth mentioning that different chemicals are found in plants, which sometimes may be hazardous as causing corrosion and irritations to the skin [18]. For this reason, toxicity studies are very important in establishing the safety of plant use. This study therefore investigates the dermal toxicity and wound healing properties of root bark of *C. ferruginea.*

## 2. Materials and Methods

### 2.1. Plant Collection

Collection of the roots of *C. ferruginea* was conducted on the campus of University of Cape Coast (Ghana post location: CC-140-7474, William Amo Road) in the Central Region of Ghana between October and November 2018. The plant was authenticated by Mr. Asare, a botanist at the Department of Herbal Medicine, Faculty of Pharmacy and Pharmaceutical Sciences (FPPS), Kwame Nkrumah University of Science and Technology (KNUST), Kumasi. A herbarium specimen (KNUST/HM1/2018/R005) was kept in the herbarium of the same department.

### 2.2. Preparation of Methanolic Extract

The roots were washed thoroughly under running tap water and dried at 28°C for two weeks. They were then cut into pieces and milled thereafter into a coarse powder using a mechanical grinder. Three hundred (300) grams of the powdered root bark were weighed, and 3 L of methanol was gently added. This mixture was swirled and left to stand at room temperature (25°C) with intermittent shaking for 72 h. The mixture was then filtered through Whatman filter papers (number 1). The filtrate was evaporated to dryness using a hot air oven at 40^o^C to a constant dry mass. The extract obtained was labeled and kept in a desiccator at 28^o^C for later use and was named *C. ferruginea* methanol extract (CFM).

### 2.3. Experimental Animals

Adult Sprague Dawley rats (150–200 g) between 8 and 10 weeks of age were obtained from the University of Ghana Animal House, Accra, Ghana. They were kept in stainless steel cages at the animal house, Department of Pharmacology, KNUST, under ambient temperature (25°C), light, and relative humidity (55 to 60%). They were fed with rat feed (GAFCO, Tema, Ghana) and water *ad libitum* throughout the experiments.

### 2.4. Ethical Approval

Rats were handled in accordance with guidelines for care and use of laboratory animals laid down by the National Institute of Health (NIH, Department of Health and Human Services Publication No. 5, Revised 1985). All animal studies in this research were consented to by the Animal Ethical Committee with the ethical clearance number FPPS-AEC/CA07/19 at the Faculty of Pharmacy and Pharmaceutical Sciences, Kwame Nkrumah University of Science and Technology, Kumasi, Ghana.

### 2.5. Toxicity Studies

#### 2.5.1. Skin Irritation Test

Ten male Sprague Dawley rats (weighing 250–300 g) were used for the skin irritation test as described by the OECD guidelines 404 [19]. The animals were put into two groups of five animals each. Five rats were used as test rats (Tr), whereas five other rats were used as control rats (Cr). Hair from the back of the rats was clipped with a sterile razor towards the lower mid-position to about 20 cm in diameter and caged individually. The rats were left undisturbed for 24 h. A volume of 0.2 ml of water was added to 0.5 g of CFM and applied evenly to the shaved site on the test rats. The plant extracts were held in contact with the skin with gauze and a nonirritant adhesive tape for 1 h, after which it was removed and the surface of the skin was rinsed with distilled water and examined for skin irritation. Observation of the sites was also done at 24 h after application and repeated at 48 and 72 h. Likewise, the control rats received a topical application of sterile water (sterile cotton soaked with sterile water), which was also secured with a gauze and nonirritant tape. Observation of oedema and erythema was made and scored according to the Draize scoring system (Table 1) [20].

#### 2.5.2. Acute Dermal Toxicity Test (Limit Test)

This experiment was performed in accordance with the OECD guidelines [19]. Ten male Sprague Dawley rats with weights between 250 and 300 g were used for this test. About 10% of the entire body surface area on the dorsal positions of the rats were shaved with a razor blade. The animals were left undisturbed for 24 h in their clean cages. The rats were grouped into two groups of five rats each (test groups and a control group). Based on the OECD guideline 404, a limited test at one dose level should be at 2000 mg/kg body weight. As a result, CFM was applied at a dose of 2000 mg/kg to shaved sites on the test group rats after 24 h. The control group received the topical application of sterile water. These were secured with gauze and a nonirritant adhesive tape. The coverings were taken off meticulously after 24 h post-application, cleaned thoroughly with distilled water, and observed for erythema or oedema. The application was done 24 hours for 14 days in both test and control groups with a daily observation for clinical changes and weekly re-weighing of rats [21]. The oedema and erythema scores were done based on the Draize dermal irritation scoring system. The weights of the animals were taken weekly. The liver, spleen, kidney, heart, brain, and lungs were weighed after the study period.

#### 2.5.3. Repeated Dose Dermal Toxicity Test

Hair occupying about ten percent of the entire back region of rats was shaved off each of the twenty experimental rats. The rats weighed between 250 and 300 g and were randomized into 4 groups of 5 rats each. These 4 groups consisted of 3 treatment groups, which used extract concentrations of 1000 mg/kg body weight (group 1), 500 mg/kg body weight (group 2), and 250 mg/kg body weight (group 3), and a control group, which employed the application of sterile distilled water only (group 4). A limit dose of not exceeding 1000 mg/kg body weight has been set as the maximum dose for this experiment as per the OECD guidelines [22]; hence, 1000 mg/kg body weight was employed as the highest test dose. The rats were then left untroubled for 24 h before the start of extract application. The extracts were applied to the shaved sites and kept in place with gauze and a nonirritating adhesive tape. The rats were treated with the extracts 12 hours for 21 days. Observation for clinical changes and weekly re-weighing was conducted [21]. Serum biochemistry, weights of internal organs (spleen, heart, liver, kidney, brain, and lungs), and histology of skin tissues were evaluated after the 21 days.

*(1) Serum Biochemistry*. Blood samples were obtained as rats bled by the cardiac puncture method after being anaesthetized with pentobarbitone at a dose of 40 mg/kg body weight. This was used in the determination of haematology parameters using potassium EDTA as an anticoagulant. Measurements of clinical biochemical parameters such as albumin, alkaline phosphatase (ALP), aspartate transaminase (AST), alkaline transaminase (ALT), gamma-glutamyl transferase (GGT), and indirect bilirubin (IND BIL) were made using a chemistry analyser (VITROS 5600) [23].

*(2) Histological Examination*. Tissues were excised from wounds on day 14 post-injury and immediately fixed in 10% buffered neutral formalin (Sigma-Aldrich, Michigan, USA). This was followed by tissue processing in an automatic tissue processor (Leica TP1020, Boston Laboratory Equipment, Boston, USA) and programmed to run a series of treatment by immersing the tissues in ethanol, xylene, and paraffin (Merck BDH, Poole, United Kingdom). The processed tissues were then cut into 5 mm sections with a Leica rotary microtome (Boston Laboratory Equipment, Boston, USA) and thereafter stained with haematoxylin and eosin (Sigma-Aldrich, St. Louis, Missouri, USA) after the paraffin was removed. The tissues were then examined with light microscope (Leica Microsystems, Wetzlar, Germany). Photomicrographs were taken at x40 magnification. The tissues were observed for the degree of cell repair, re-epithelialization, angiogenesis, collagen content, and granular tissue formation. Verhoeff–Van Gieson's stain (Sigma-Aldrich, St. Louis, MO, USA) was also employed to evaluate the level of collagen formation, deposition, and organization [24].

### 2.6. Wound Healing Studies Using Excision Wound Model

Thirty Sprague Dawley rats (120–200 g) were used for this investigation. The rats were placed in groups of five in a total of six groups. The animals were anaesthetized with pentobarbitone at a dose of 40 mg/kg body weight. The dorsal furs of the animals were clipped in a circular manner to a diameter of about 50 mm by means of a razor blade and cleaned with 70% ethanol. The borders of the wound were outlined using a marker filled with ammonium oxalate violet paint on the shaved skin of the rats. The rats were inflicted with excision wounds along the markings using sterile toothed forceps, surgical blades, and pointed scissors to a diameter of 10 mm. The wounds were left open, and the animals were placed in cages with five rats in each cage [8]. The aqueous creams used for the wound healing experiment were prepared according to the method described in the British Pharmacopoeia (BP) [25]. The preservative was discarded from the preparation to prevent its interference with the wound healing activity of the extracts. One hundred grams of aqueous cream was prepared by mixing 30 g of emulsifying ointment in 70 mL of sterile distilled water. The mixture was heated in a water bath and stirred in one direction until a homogenous mixture was obtained. This mixture was allowed to cool. The extract was then incorporated into the homogenous cream separately to form three different concentrations of the extract creams at 2.5, 5, and 10% w/w. The physical stability of the creams was monitored for phase separation, colour, odour, and texture. Wound treatment began 24 h post-injury and lasted for 14 days.

- Group 1: rats were treated with 10% w/w CFM aqueous cream
- Group 2: rats were treated with 5% w/w CFM aqueous cream
- Group 3: rats were treated with 2.5% w/w CFM aqueous cream
- Group 4: rats were treated with 1% w/w silver sulphadiazine (SSD) as a positive control
- Group 5: rats were treated with the blank aqueous cream only
- Group 6: rats were left untreated (wounds cleaned with normal saline daily)

#### 2.6.1. Assessment of Wound Diameter

The diameters of the wounds were measured every other day beginning from the wound creation day (Day 1) until Day 14 with a measuring rule. The contraction of the wounds based on size was recorded accordingly and calculated as a percentage with the following equation:

(1) $$\begin{array}{c} \%\text{ of wound contraction}=\frac{\left(\text{initial wound size}-\text{specific day wound size}\right)\times 100}{\text{Initial wound size}}. \end{array}$$

#### 2.6.2. Histology Studies

This was carried out using the same procedure as described in section 2.5.3.2.

### 2.7. Phytochemical Screening

The root bark extract of C. ferruginea was screened for the presence or absence of secondary metabolites such as tannins, glycosides, terpenoids, saponins, flavonoids, alkaloids, steroids, and coumarins. The phytochemical tests performed are summarized in Table 2.

### 2.8. Data Analysis

The data obtained for the tests carried out were statistically analysed with GraphPad Prism version 5.0 (GraphPad Software, San Diego, CA, USA), and results were presented as the mean ± standard error mean (SEM). A two-way ANOVA followed by the Bonferroni *post hoc *test was used to analyse the anti-inflammatory and wound healing time-course curves. The area under the curve (AUC) was analysed using the one-way ANOVA followed by Dunnett's *post hoc* test.

## 3. Results

### 3.1. Toxicity Studies

**Figure 1:**
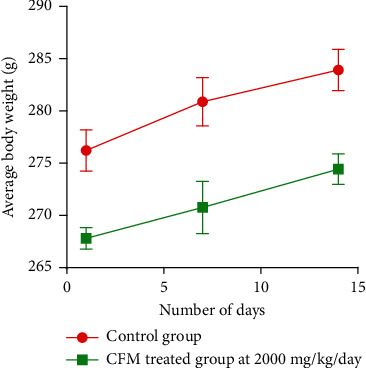
Weekly average weight of rats over 14-day study period.

In the skin irritation test, no erythema or oedema was seen in both the control animals and the test animals after 3 days of experimentation. In determining the dermal toxic effect of CFM in acute dermal toxicity bioassay, no significant (*P* > 0.05) clinical change was observed in any of the treated rat groups except for the initial reaction within the first 30 mins of patch attachment when the rats tried to tear the patch off. The behavioral patterns and general appearance of the rats in the control and test groups were recorded after one hour and twelve hours post-application of test substances. No change meant that the manner in which the animals behaved after acclimatization did not alter when the skin was shaved and test substances were applied. No erythema or oedema was observed over the 14-day study period in both the control and CFM-treated animals. The individual weights of the rats over the study period progressed steadily showing no outliers (Figure 1). There was no significant (*P* > 0.05) change in the relative organ weight of the CFM-treated group compared with the control group (Table 3).

**Figure 2:**
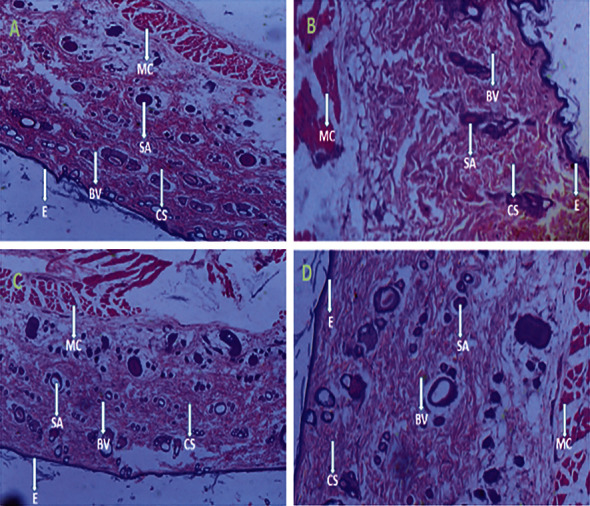
Histology of skin tissues after 21-day toxicity study period. (a) Skin tissue of control animal, (b) skin tissue of CFM-treated rat at 250 mg/kg, (c) skin tissue of CFM-treated rat at 500 mg/kg, and (d) skin tissue of CFM-treated rat at 1000 mg/kg. Picture note. CS: collagen strands; BV: blood vessels; SA: skin appendages; E: epithelium; MC: muscle cell.

In determining the dermal toxic effect of CFM in the repeated dose dermal toxicity bioassay, observation of the rats over the entire study period showed no toxic effect. Rats tried to remove the patch within the first 30 mins of patch attachment; however, no erythema or oedema was observed over the 21-day study period in both the control and CFM-treated animals. The maximum limit dose for a repeated dose toxicity test is recommended not to exceed 1000 mg/kg. Two other dose levels were proportionally set to be 500 mg/kg and 250 mg/kg. An increase in weight was observed within all the study groups in a similar fashion (Table 4). No rat died within the study period. There was no significant (*P* > 0.05) change in the average weight and relative organ weight of the CFM-treated group compared with the control group (Tables 4 and 5). In the haematological and serum biochemical analysis, the analysis showed similar results between the control animals and CFM-treated animals in the parameters, which were investigated (Tables 6 and 7). Skin tissue histology was performed on skin tissues of the rats from both CFM-treated and control groups. Observed from the tissues were collagen strands, blood vessels, skin appendages, epithelium, and muscle cells. No pus cells or ruptured blood vessels were observed (Figure 2).

### 3.2. Wound Healing Activity of CFM

#### 3.2.1. Influence of CFM on Wound Contraction

**Figure 3:**
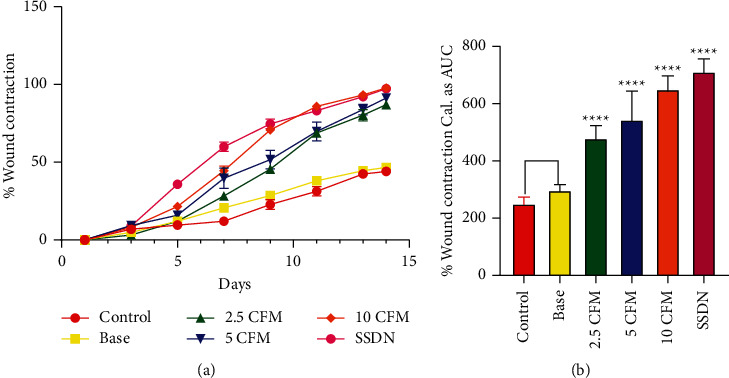
Influence of CFM on the rate of contraction of excision wounds. (a) Time-course curve of CFM on wound contraction expressed as percentage. (b) AUC of percentage wound contraction.

The effect of CFM on the excision wounds of Sprague Dawley rats was evaluated by taking intermittent measurements over the 14 days on alternate days (Figure 3(a)). Compared with the untreated group, 2.5, 5, and 10% w/w aqueous creams of CFM-treated wounds significantly reduced the wound size over the study period (*P* < 0.0001). The blank cream (base), however, showed no significant effect on wound contraction compared with the untreated group (*P* = 0.5029). The silver sulphadiazine-treated group (positive control) showed a significant reduction in the wound size over the study period (*P* < 0.0001) compared with the untreated group (Figure 3(b)).

#### 3.2.2. Histological Evaluation of CFM-Treated Wound Tissues

**Figure 4:**
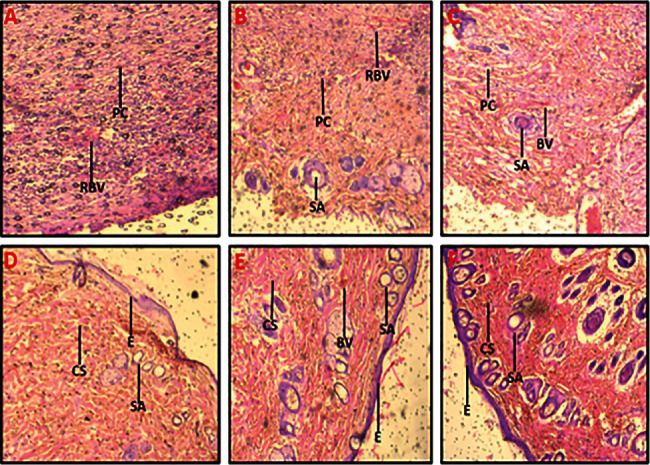
Histological images (x40) from the wound healing activity. (a) Untreated wound tissues, (b) base (aqueous cream only)-treated tissues, (c) 2.5% w/w CFM-treated wounds tissues, (d) 5% w/w CFM-treated wounds tissues, (e) 10% w/w CFM-treated wound tissues, and (f) 1% w/w silver sulphadiazine-treated wound tissues. Picture note. CS: collagen strands; BV: blood vessels; RBVs: ruptured blood vessels; PCs: pus cells; SA: skin appendages; E: epithelium.

The wound tissues after the study period of 14 days were excised and evaluated histologically by staining the tissues with haematoxylin and eosin. Neovascularization, epithelial regeneration, and collagen deposition were looked out for in the evaluation. Tissue slides were read at a magnification of 40 and represented in Figure 4.

Untreated wound tissues revealed massive necrosis of the skin tissues, which is indicative of poor wound healing (Figure 4(a)). Base-treated wound tissues (aqueous cream only) were also observed to be inflamed. Pus cells were seen all over the epidermal layer with ruptured blood vessels. A poor healing process occurred (Figure 4(b)). In the CFM-treated (2.5% w/w) wound tissues, skin tissues are comprised of the moderately thinned epidermis with moderate residual inflammatory cells and ruptured blood vessels. A moderately healed wound was observed (Figure 4(c)). CFM-treated (5% w/w) wound tissues: there was increased collagen formation. No oedematous skin appendages were observed. These indicated good wound healing activity (Figure 4(d)). In CFM-treated (10% w/w) wound tissues, skin tissues were observed to have normal skin appendages and well-formed blood vessels. The hair follicles and the underlying fatty layer were normal, indicative of well-healed wounds (Figure 4(e)). Silver sulphadiazine-treated (1%) wound tissues showed normal skin appendages and well-formed blood vessels. Wound edges appeared normal, which was indicative of good wound healing activity (Figure 4(f)).

### 3.3. Phytochemical Screening

The qualitative assessment for the secondary metabolites of the root bark extract of *C. ferruginea* was undertaken to indicate the presence or absence of secondary metabolites such as tannins, glycosides, terpenoids, saponins, flavonoids, alkaloids, steroids, and coumarins. The phytochemical composition of *C. ferruginea* root bark extract is shown in Table 8.

## 4. Discussion

### 4.1. Dermal Toxicity of CFM

Skin irritation involves local inflammation, which presents as erythema and oedema following direct skin injury [30]. This direct skin injury may be a single, repeated, or prolonged contact with chemical substance to the skin [31]. The rat model, which is a well-established model for dermal toxicity studies [32–35], was used in this study as they were readily available and would offer easy comparative studies as the subsequent predictive studies in this research used rats as the preferred model for study. Again, rats are known to respond to therapies and their toxic effects in a way similar to humans hence can be used to predict the likelihood of a potential toxicity of an agent when administered to humans [36]. The skin irritation test revealed that CFM was not an irritant as no erythema and oedema were observed in the test group at all the times employed in the experiment. The erythema scores and the oedema scores were all zero (0).

The acute dermal toxicity studies were performed according to the OECD guidelines. This test is often a stepping stone for long-term toxicity tests as it sets the suitable dose for use in such studies and provides a gist of the dose-response relationship. Again, it reveals the most affected organ in the study, and if any is found, it provides a basis for better designing of long-term studies [23]. The animals showed no significant clinical change. The behavior of the control and the test-treated animals was alike. No oedema or erythema was observed over the entire period of study. Toxic agents may affect major organs in the body, thus impairing their physiological functions. For this reason, the internal organs were assessed by way of their weights, which may give a prior idea about some physiological happenings to the body. The weights of the internal organs to the body ratio of the animals as expressed in relative weights showed similarities between the CFM-treated animals and the control group animals in the study. This shows that the extract did not exhibit any deviating effect on the internal organs of the rats. Organ weight changes, that is, atrophy and hypertrophy, have long been accepted as a sensitive indicator of chemically induced changes to organs. In toxicological experiments, a comparison of organ weights between treated and untreated groups of animals has conventionally been used to evaluate the toxic effect of the test article [37, 38]. According to Nirogi et al. [39] changes in organ weights are predictive of the mechanism of toxicity instead of histopathology and therefore should be augmented with biochemical and haematological parameters. The individual weights of the rats progressed steadily over the study period in all study groups. The repeated dose dermal toxicity test followed the acute dermal toxicity test. In this study, the assessment of toxicity was followed by haematology and serum biochemistry studies. Platelets were significantly elevated in 1000 mg/kg CFM-treated rats (836 ± 14.1(10^3^/*μ*L); 864 ± 15.2(10^3^/*μ*L)) compared with the control group (*P* = 0.02). All other parameters did not show significant differences between the control and test groups. In reference to the sub-chronic test conducted by Ishola et al. [40], CFM may possibly have an effect on the formation of platelets and must be used with caution over long periods. Thus, upon dermal usage, the extract may have a toxic assault at very high concentrations upon repeated use. However, it is difficult to declare this plant as toxic due to this very one outlier unless upon further studies. In toxicity studies, after chemical analyses and observations, it is usually important to investigate the histopathologic outcome of the target organs or the organ under study for a better conclusion and evaluation of the chemical agent under study [41]. The skin, being the target organ under study, revealed intact epidermal layers comprising normal skin appendages and dermis. The hair follicles and the sebaceous glands as well as other skin appendages all remained normal in the control and treatment groups. No inflammatory cells or ruptured blood vessels were observed in any group. The dermis also revealed dense collagen. The impact of CFM on the skin is thus safe.

### 4.2. Wound Healing Activity of CFM

Wound healing is a natural occurrence, which deals with tissue regrowth and regeneration [42]. This study showed that the treatment of excised wounds of Sprague Dawley rats with aqueous creams of the methanol extract of the root bark of *C. ferruginea* accelerated the wound healing process. Yakubu et al. [12] have also reported that the hydroethanolic leaf extract of *C. ferruginea* exhibits wound healing activity. All the three extracts used, significantly (*P* < 0.0001) promoted wound healing (measured as rate of wound contraction) as compared to the control. Wound contraction is the movement of the edges of a full-thickness wound towards the midportion to ensure the closure of the injury [43].

Microorganisms play an inevitable role in wounds by colonizing them; thus, every wound stands the risk of getting infected. These microorganisms may produce chemicals, which prolong the inflammatory stage of wound healing by the production of various enzymes [44]. Again, some of these attacking microorganisms may form biofilms on the wounds. These biofilms act as physical barriers, which prevent the penetration of antimicrobials. This propels the wounds into chronic stages as there remains an accumulation of damaged tissues and proteins [45]. Plant extracts with antimicrobial activity have also been reported to promote wound healing since infected or colonized wounds usually delay the healing process and sometimes lead to chronic wounds [8]. Hence, the reported antimicrobial activity of *C. ferruginea* [46] may contribute to its wound healing activity Also, subinhibitory concentrations of the methanolic extract of *C. ferruginea*, tested for their ability to inhibit biofilms in four microorganisms namely *P. aeruginosa*, *E. coli*, *S. aureus,* and *K. pneumoniae,* were able to significantly reduce the formation of biofilms, *P* < 0.0001 in a study by Ankomah et al. [46]. This is an indication that *C. ferruginea* may be a very good choice in managing wounds as it will be able to prevent or decrease the formation of biofilms in microorganisms, which usually makes the management of wounds challenging. The antioxidant and anti-inflammatory properties of *C. ferruginea* were also reported by Akharaiyi et al. [16]. These properties of the plant may have conferred the observed wound healing properties on the plant as compared to the groups, which were not treated with extracts. The observation, however, revealed that this wound healing property of the plant was dose-dependent with respect to the concentrations studied. The untreated group in comparison with the base-treated group did not show any significant increase in wound contraction. Collagen is one of the most abundant proteins of extracellular matrix [47]. Not only do they provide structural support to the skin but also control some cellular functions such as cell shape, differentiation, migration, and synthesis of a number of other proteins [48]. Also, collagen ensures the migration of endothelial cells to form new blood vessels to help granulate tissue formation, which eventually improves wound healing and is observed as a decrease in the wound area or wound contraction [49]. Fibroblasts also play a vital role in the formation of the granulation tissue, which moves mainly from the nearby dermis to the wound in response to cytokines and growth factors [50]. It is also worth mentioning that after wounds are created, the inflammatory stage begins to clear the wound of dead cells and microbes. This induces the creation of a dense but poorly organized capillary bed. During the angiogenic phase, most of these newly formed vessels are pruned, creating a final vessel density that is similar to that of normal skin [51].

Histology of the wound tissues revealed a well-formed skin in rats treated with 10% w/w CFM cream and the rats treated with 1% w/w silver sulphadiazine cream with no residual inflammatory cells and normal blood vessels and normal extracellular matrix indicating no sign of excessive wound healing. The histology also revealed that the untreated and base-treated groups had very poorly healed wounds due to massive necrosis of skin. 2.5% and 5%w/w CFM-treated rats had mild to moderate collagenization and fibrous tissue formation and moderate residual inflammatory cells. CFM thus ensured proper wound healing with the highest study dose of 10% w/w of the extract, showing the best activity.

The findings of this study give credence to the folkloric use of *C. ferruginea* in the treatment of wounds. Additionally, this study has shown that the plant extract is safe for use topically, which may partly confirm why the plant is still used in the management of wounds. Secondary metabolites possessed by plants are responsible for the pharmacological effects they exhibit [52]. The observed wound healing activity of *C. ferruginea* root extract may be due to the presence of phytochemicals such as flavonoids, tannins, saponins, and triterpenoids that the plant possesses (Table 8). Flavonoids prevent the onset of cell necrosis and improve the flow of blood to the wounded site [53]. Again, flavonoids and tannins are well known to have antioxidant, astringent, and antimicrobial activities, which are essential for wound healing [54]. Tannins also have the ability to promote fibroblast proliferation and initiate its migration into wounds [55]. Likewise, saponins have also been reported to contain antibacterial properties [56]. Saponins from plant studies have been reported to enhance wound healing activity [57, 58] and prevent excessive scar formation in experimental mice [59]. Triterpenoids are also phytochemicals, well known to have anti-inflammatory and antibacterial activities [60].

## 5. Conclusion

*C. ferruginea* methanol root extract exhibited wound healing activity. Wound closure rate revealed that 10% w/w CFM-treated wounds showed the best wound healing activity as compared to 5% and 2.5% w/w CFM-treated rats, which showed moderate wound healing activity. Tissue histology revealed a healed wound with well-regenerated collagen and skin appendages with no pus cells. CFM also showed no toxic effect on the skin. The wound healing activity of *C. ferruginea* gives credence to its folkloric use. However, the efficacy of the root extract should be evaluated in other wound models.

## Figures and Tables

**Table 1:** Draize dermal irritation scoring system.

Score	Translation
0	No erythema or oedema
1	Very inappreciable oedema or erythema
2	Small oedema with raised skin at the edges of the area
3	Moderate to severe erythema or oedema
4	Severe erythema or oedema

**Table 2:** Phytochemical test performed on the root bark extract of *C. ferruginea*.

Secondary metabolite	Test performed	Reference
Tannins	Ferric chloride test	Boakye et al. [26] Evans, [27]
Saponins	Foaming/frothing test	Boakye et al. [26] Evans, [27]
Glycosides	Fehling's test	Evans, [27] Gul et al. [28]
Terpenoids	Acetic anhydride test	Evans, [27] Shaikh and Patil [29]
Steroids	Libermann–Burchard's test	Shaikh and Patil [29] Evans, [27]
Alkaloids	Dragendorff's/Kraut's test	Boakye et al. [26] Evans, [27]
Flavonoids	Lead acetate test	Shaikh and Patil [29] Evans, [27]
Coumarins	NaOH paper test	Shaikh and Patil [29] Evans, [27]

**Table 3:** Relative organ weight of rats after the 14-day study period.

Organs	Relative organ weight	
	Control	2000 mg/kg/day
Kidney	0.88 ± 0.03	0.89 ± 0.02
Liver	3.47 ± 0.02	3.46 ± 0.04
Brain	2.42 ± 0.03	2.45 ± 0.03
Spleen	0.33 ± 0.01	0.32 ± 0.02
Heart	0.46 ± 0.02	0.46 ± 0.03
Lungs	0.80 ± 0.04	0.79 ± 0.04

Values expressed as mean ± SEM, *n* = 5 female rats. Comparison of CFM at 2000 mg/kg/day to control indicates significance levels of *P*=0.25 (Wilcoxon matched-pairs signed-rank test).

**Table 4:** Average weight of rats over 21-day study period.

Group	Weights (g) of rats over 21-day study period						
	Week 0	*P* value	Week 1	*P* value	Week 2	*P* value	Week 3
Control	275.37 ± 8.42	**>0.99**	277.57 ± 8.94	**>0.99**	280.30 ± 9.41	**>0.99**	282.43 ± 9.81
TG-1	272.41 ± 8.17	**>0.99**	274.06 ± 8.88	**>0.99**	275.51 ± 9.37	**>0.99**	279.06 ± 7.46
TG-2	275.26 ± 11.23	**>0.99**	276.62 ± 11.83	**>0.99**	278.65 ± 12.04	**>0.99**	281.37 ± 11.33
TG-3	277.61 ± 9.97	**>0.99**	279.77 ± 9.81	**>0.99**	281.84 ± 9.82	**>0.99**	283.94 ± 10.02

TG-1: 250 mg/kg/day, TG-2: 500 mg/kg/day, TG-3: 1000 mg/kg/day, TG: CFM-treated group, CG: control group. Values expressed as mean ± SEM, *n* = 10 (one-way ANOVA).

**Table 5:** Relative organ weight of rats treated with different doses of extract for 21 days.

Organs	Control	Relative organ weight of CFM-treated rats					
		250 mg/kg	*P* value	500 mg/kg	*P* value	1000 mg/kg	*P* value
Kidney	0.87 ± 0.03	0.86 ± 0.03	**0.73**	0.87 ± 0.03	**>0.99**	0.89 ± 0.01	**0.23**
Liver	3.46 ± 0.02	3.44 ± 0.01	**0.19**	3.45 ± 0.02	**0.7**	3.44 ± 0.04	**0.19**
Brain	2.45 ± 0.03	2.43 ± 0.05	**0.43**	2.44 ± 0.03	**0.85**	2.45 ± 0.02	**>0.99**
Spleen	0.31 ± 0.01	0.31 ± 0.01	**>0.99**	0.31 ± 0.01	**>0.99**	0.32 ± 0.02	**0.23**
Heart	0.45 ± 0.03	0.44 ± 0.02	**0.71**	0.44 ± 0.02	**0.71**	0.45 ± 0.03	**>0.99**
Lungs	0.81 ± 0.01	0.80 ± 0.02	**0.85**	0.79 ± 0.04	**0.42**	0.79 ± 0.05	**0.42**

Values expressed as mean ± SEM, *n* = 10 (one-way ANOVA).

**Table 6:** Haematological parameters for the toxicity test.

Test	Control	CFM					
		250 mg/kg	*P* value	500 mg/kg	*P* value	1000 mg/kg	*P* value
RBCs (10^6^/*μ*L)	8.10 ± 0.6	8.12 ± 0.5	**>0.99**	8.0 ± 1.1	**0.99**	7.94 ± 1.1	**>0.96**
HB (g/dL)	14.5 ± 1.2	14.3 ± 1.1	**0.97**	13.9 ± 1.6	**0.61**	13.8 ± 1.3	**0.50**
HCT (%)	47.2 ± 1.1	47.2 ± 0.6	**>0.99**	46.9 ± 1.2	**0.93**	47.5 ± 2.1	**0.93**
MCV (*μ*m^3^)	58.3 ± 2.3	58.3 ± 3.3	**>0.99**	58.1 ± 1.0	**0.99**	57.1 ± 1.6	**0.42**
MCH (pg)	17.6 ± 0.7	18.2 ± 0.1	**0.58**	17.4 ± 1.2	**0.97**	17.2 ± 2.1	**0.82**
MCHC (g/dL)	30.7 ± 1.4	30.7 ± 1.4	**>0.99**	29.9 ± 1.0	**0.41**	32.4 ± 1.5	**0.79**
Platelets (10^3^/*μ*L)	836 ± 14.1	835 ± 14.1	**0.99**	843 ± 12.1	**0.54**	864 ± 15.2	**0.04 ** ^*∗*^
WBCs (10^3^/*μ*L)	9.4 ± 3.1	9.4 ± 3.2	**>0.99**	8.78 ± 0.9	**0.96**	8.9 ± 5.3	**0.98**

RBCs: red blood cells, HB: haemoglobin, HCT: haematocrit, MCV: mean corpuscular volume, MCH: mean corpuscular haemoglobin, MCHC: mean corpuscular haemoglobin concentration, WBCs: white blood cells. Level of significance: values expressed as mean ± SEM, *n* = 10 (one-way ANOVA).

**Table 7:** Biochemical analysis of the serum.

Test	Control	CFM					
		250 mg/kg	*P* value	500 mg/kg	*P* value	1000 mg/kg	*P* value
Albumin (g/dL)	21.5 ± 1.7	21.5 ± 1.8	**>0.99**	22.6 ± 2.1	**0.47**	22.7 ± 2.3	**0.40**
ALP (U/L)	198.4 ± 0.8	197.4 ± 0.5	**0.13**	198.3 ± 1.6	**0.99**	198.0 ± 1.2	**0.76**
AST (U/L)	353.3 ± 1.4	353.3 ± 1.6	**>0.99**	354.5 ± 2.9	**0.41**	353.6 ± 1.8	**0.98**
ALT (U/L)	130.6 ± 2.2	131.6 ± 2.1	**0.52**	129.2 ± 2.1	**0.26**	129.0 ± 1.1	**0.45**
GGT (U/L)	<6.0	<6.0	**>0.99**	<6.0	**>0.99**	<6.0	**>0.99**
IND BIL (*μ*mol/L)	1.3 ± 0.01	1.4 ± 0.1	**0.99**	1.3 ± 0.02	**>0.99**	1.2 ± 1.3	**0.99**

ALP: alkaline phosphate, AST: aspartate transaminase, ALT: alanine transaminase, GGT: gamma-glutamyl transferase, IND BIL: indirect bilirubin. Values expressed as mean ± SEM, *n* = 10 (one-way ANOVA).

**Table 8:** Phytochemical composition of CFM.

Secondary metabolite	CFM
Tannins	+
Glycosides	+
Saponins	+
Flavonoids	+
Coumarins	+
Steroids	−
Terpenoids	+
Alkaloids	+

+presence of phytochemical, − absence of phytochemical.

## Data Availability

The datasets used and/or analysed during this study are included within the article. Further clarification can be obtained from the corresponding author.
